# Alterations in local thyroid hormone signaling in the hippocampus of the SAMP8 mouse at younger ages: Association with delayed myelination and behavioral abnormalities

**DOI:** 10.1002/jnr.23161

**Published:** 2013-03

**Authors:** Erika Sawano, Takayuki Negishi, Tomoyuki Aoki, Masami Murakami, Tomoko Tashiro

**Affiliations:** 1 Department of Chemistry and Biological Science, Aoyama Gakuin UniversitySagamihara, Kanagawa, Japan; 2 Department of Clinical Laboratory Medicine, Gunma University Graduate School of MedicineMaebashi, Japan

**Keywords:** thyroid hormone, SAMP8, type 2 deiodinase, hippocampus, hyperactivity, myelin basic protein

## Abstract

The senescence-accelerated mouse (SAM) strains were established through selective inbreeding of the AKR/J strain based on phenotypic variations of aging and consist of senescence-prone (SAMP) and senescence-resistant (SAMR) strains. Among them, SAMP8 is considered as a model of neurodegeneration displaying age-associated learning and memory impairment and altered emotional status. Because adult hypothyroidism is one of the common causes of cognitive impairment and various psychiatric disorders, we examined the possible involvement of thyroid hormone (TH) signaling in the pathological aging of SAMP8 using the senescence-resistant SAMR1 as control. Although plasma TH levels were similar in both strains, a significant decrease in type 2 deiodinase (D2) gene expression was observed in the SAMP8 hippocampus from 1 to 8 months of age, which led to a 35–50% reductions at the protein level and 20% reduction of its enzyme activity at 1, 3, and 5 months. D2 is responsible for local conversion of thyroxine into transcriptionally active 3,5,3′-triiodothyronine (T3), so the results suggest a reduction in T3 level in the SAMP8 hippocampus. Attenuation of local TH signaling was confirmed by downregulation of TH-dependent genes and by immunohistochemical demonstration of delayed and reduced accumulation of myelin basic protein, the expression of which is highly dependent on TH. Furthermore, we found that hyperactivity and reduced anxiety were not age-associated but were characteristic of young SAMP8 before they start showing impairments in learning and memory. Early alterations in local TH signaling may thus underlie behavioral abnormalities as well as the pathological aging of SAMP8. © 2012 Wiley Periodicals, Inc.

The senescence-accelerated mouse (SAM) strains were established through selective inbreeding of the AKR/J strain based on phenotypic variations of accelerated aging, such as amyloidosis, osteoporosis, and learning and memory deficits (Takeda et al., 1981). They consist of senescence-prone (SAMP) strains, which exhibit accelerated aging with a shorter life span, and senescence-resistant (SAMR) strains, which show normal aging. Among the SAMP strains, the SAMP8 strain displays mainly age-associated impairments in CNS function, including marked deficits in learning and memory (Miyamoto et al., 1986; Flood and Morley, 1992, 1993; Yagi et al., 1998), altered emotional status (Miyamoto et al., 1992; Markowaska et al., 1998), and abnormality of circadian rhythm (Miyamoto et al., 1986; Colas et al., 2005), with relatively mild physical impairments. Neuropathological and neurochemical studies have further shown that pathological changes observed in the aged brain such as increased oxidative stress, occurrence of Aβ-immunoreactive deposits (Del Valle et al., 2010), and neuroinflammation are detected earlier and at increased severity in the SAMP8 brain, especially in the hippocampus, confirming the usefulness of this strain as a model for neurodegeneration (for review see Butterfield and Poon, 2005; Takeda, 2009; Tomobe and Nomura, 2009). The causes for such accelerated senescence, however, remain to be elucidated.

We focused on the thyroid hormone (TH) signaling as one potential contributing factor to accelerated aging in SAMP8, because TH plays essential roles not only in the developing brain (Oppenheimer and Schwartz, 1997; Koibuchi and Chin, 2000; Bernal, 2005; Williams, 2008) but also in the mature brain to maintain its proper functions. Adult hypothyroidism is one of the most common causes of transient dementia, and thyroid disorders have been linked to various psychiatric and neuropsychological disorders, including learning deficits, impaired attention, anxiety, and depression (for review see Davis and Tremont, 2007).

TH exerts its effects mainly through binding to the specific nuclear receptors of the steroid-retinoic acid-TH receptor superfamily, which function as ligand-regulated transcription factors (Oppenheimer and Schwartz, 1997; Koibuchi and Chin, 2000; Williams, 2008). The transcriptionally active form of TH that binds to these receptors is 3,5,3′-triiodothyronine (T3) produced by enzymatic deiodination of the initially synthesized form, thyroxine (T4). In addition to the well-known feedback system consisting of hypothalamus, pituitary, and thyroid gland (the HPT axis), which regulates the production of T4, it is now established that the concentration of T3 is locally regulated by three types of iodothyronine deiodinases; type 1 and 2 deiodinases (D1, D2), which convert T4 into active T3, and type 3 deiodinase (D3), which inactivates T3 (Gereben et al., 2008; Dentice and Salvatore, 2011). The T3-producing enzyme in the CNS is D2, which is expressed in astrocytes, whereas D3 is expressed in neurons.

In the present study, we examined the expression of D2 and D3 as well as representative TH-dependent genes to evaluate the local T3 availability in the hippocampus of SAMP8. The results indicate that, in spite of normal plasma TH level, the local TH signaling was significantly attenuated in the SAMP8 hippocampus from an early age (1 month) because of a significant reduction in D2 activity caused by a decrease at both mRNA and protein levels. At the same time, we found that, unlike the previously described behavioral abnormalities of SAMP8, hyperactivity and reduced anxiety were not age-associated but were characteristic of young SAMP8 (1–5 months), before they start showing symptoms of learning and memory impairment. Reduced local TH signaling starting during development may thus be a cause of abnormal behavior as well as accelerated aging of SAMP8.

## MATERIALS AND METHODS

### Animals

Male SAMP8 and SAMR1 mice were purchased from SLC (Shizuoka, Japan). They were housed individually under controlled temperature (24°C ± 1°C) on a 12-hr-light (06:00–18:00 hr):12-hr-dark (18:00–06:00 hr) cycle. Food and water were available ad libitum. To determine optimal conditions for the measurement of hippocampal iodothyronine deiodinase activity, 1-month-old male ICR mice (SLC) were used. All animal treatments were approved by the Animal Experimentation Committee of Aoyama Gakuin University and were carried out under veterinary supervision and in accordance with the Society for Neuroscience Guidelines for the use of animals in neuroscience research.

For one series of experiments, six mice each of SAMR1 and SAMP8 strains were used at 1, 3, 5, 8, and 10 months of age. After the behavioral tests described below, they were sacrificed under deep ether anesthesia. Blood samples were drawn from the heart and collected with EDTA as anticoagulant. After perfusion with phosphate-buffered saline (PBS), the brains were removed and separated sagitally into halves. In total five series of animals were used to obtain tissue samples for mRNA and protein extractions, measurement of enzyme activity, and immunohistochemical observation. For mRNA and protein extractions and enzyme activity measurements, hippocampi were removed, immediately frozen in liquid N_2_, and kept at −80°C until use. For immunohistochemistry, brain halves were immediately fixed in 4% paraformaldehyde (PFA).

### Passive Avoidance Test

Learning and memory abilities of SAMP8 and SAMR1 mice were examined at 1, 3, 5, 8, and 10 months by passive avoidance test using a two-compartment (light and dark) step-through cage (Muromachi Kikai Co., Tokyo, Japan). The tests were conducted between 6:00 and 10:00 PM. On the first day of examination (day 1), each mouse was placed in the light compartment, and the latency before entering the dark compartment (up to 60 sec) was measured. The mouse received an electrical shock (0.5 mA, 1 sec) immediately after entering the dark compartment. These trials were repeated up to five times until each mouse had learned to stay in the light compartment for at least 60 sec. On the next day (day 2), each mouse was placed in the light compartment, and the latency before entering the dark compartment (up to 300 sec) was measured.

### Open-Field Test

Behavior and locomotor activity of SAMP8 and SAMR1 mice were examined at 1, 3, 5, 8 and 10 months by open-field test in a rectangular field (60 cm × 90 cm with walls of 45 cm height) with which all animals were unfamiliar. The tests were conducted between 6:00 and 10:00 pm. Each mouse was placed in the center of the field, and its behavior and locomotor activity were monitored for 5 min using a video camera and computerized analysis system (Smart System; Panlab, Barcelona, Spain).

### Measurement of Plasma T4 and T3 Levels

Blood samples were centrifuged at 1,500*g* for 20 min at room temperature to obtain the plasma as the supernatant. T4 and T3 in the plasma from SAMP8 (n = 6) and SAMR1 (n = 6) were measured by competitive enzyme-linked immunosorbent assay (ELISA) kit (Diagnostic Automation Inc.) according to the protocols recommended by the manufacturer.

### Real-Time Quantitative Fluorescence-Based PCR

Total RNA was prepared individually from each hippocampus obtained from six mice of both strains at 1, 3, 5, 8, and 10 months using Trizol reagent (Invitrogen Life Technologies, Carlsbad, CA). The integrity of RNA samples was routinely monitored by microcapillary electrophoresis using a Bioanalyzer 2100 (Agilent Technologies, Palo Alto, CA). First-strand cDNA was synthesized from 1 μg total RNA from one animal using SuperScript III reverse transcription kit (Invitrogen Life Technologies). Expression levels of the following 10 genes in cDNA samples were quantified by fluorescence-based real-time PCR using Step One (Applied Biosystems, Foster City, CA) with SYBR Premix ExTaq (Takara, Shiga, Japan): cyclophilin-A (*ppia*), type 2 deiodinase (*dio2*), type 3 deiodinase (*dio3*), monocarboxylic acid transporter 8 (*mct8*), thyroid hormone receptor α (*thra*), thyroid hormone receptor β (*thrb*), neurogranine (*rc3*), hairless (*hr*), myelin basic protein (*mbp*), and ectonucleotide pyrophosphatase/phosphodiesterase 2 (*enpp2*). Cyclophilin-A (*ppia*) was used as an internal standard gene. PCR primers were designed in Oligo 6.0 primer analysis software (Molecular Biology Insights; Takahashi et al., 2008). Sequences of the PCR primers are listed in Table I.

**I:** Primers for Real-Time Quantitative PCR

	Forward	Reverse
*ppia*	GCAAGACCAGCAAGAAGATCACC	CTTCAGTGAGAGCAGAGATTACAG
*dio2*	CTACTACCTATGATCTGATTAAGTG	GGCTGGAACTAACACTTCAGTCC
*dio3*	CGACTACGCACAAGGGACCCG	CGATGTAGATGATAAGGAAGTCAAC
*thra*	CACCCCTATACACACAGAGAGC	GCCAAGCCAAGCCAAGCCAAG
*thrb*	CTATGACCCAGACAGCGAGACTC	CAGAGACATGCCCAGGTCAAAG
*mct8*	CATTCACAGGTCCCAGATCTGCCC	GCCCACACGTGCACACACGC
*rc3*	GACTTCCCTACTGTGTTTGTGAG	CTACGCCACGACGAAGCCAGC
*hr*	GCTGACCCCTCCCCTCATGG	GCAGTTGAGATACACAGAGGAAG
*mbp*	GGCACAGAGACACGGGCATCC	GCGACTTCTGGGGCAGGGAGC
*enpp2*	GTCAGAAAGGAATGGGGTCAACG	AGTGGGTAGGGACAGGAATAGAG

### Western Blotting

Each hippocampal sample obtained from SAMP8 or SAMR1 at 1, 3, 5, 8, and 10 months (n = 5–8 for either strain at each time point) was homogenized in 10 volumes of SDS sample buffer (0.06 M Tris-HCl, pH 6.8, 10% glycerol, 2% SDS, 0.04% bromophenol blue, 2% β-mercaptoethanol) containing protease inhibitors (Complete Mini EDTA-Free Protease Inhibitor Cocktail and PhosSTOP Phosphatase Inhibitor Cocktail; Roche Applied Science, Indianapolis, IN) using a glass-Teflon homogenizer, boiled at 100°C for 5 min, and centrifuged at 15,000*g* for 10 min at RT. Supernatants containing extracted proteins were collected and separated by SDS-PAGE on Tris-HCl gels followed by electrophoretic transfer onto polyvinyl difluoride (PVDF) membranes (Millipore, Bedford, MA). The blots were blocked for 1 hr at RT with 2% bovine serum albumin (BSA) or 2.5% skimmed milk in PBS containing 0.1% Tween 20 (PBS-T) and 0.02% sodium azide and incubated overnight at 4°C with one of the following primary antibodies in 2% BSA and 0.02% sodium azide-containing PBS: rabbit polyclonal anti-type 2 deiodinase (D2; 1:1,000; Abcam, Cambridge, United Kingdom), rabbit polyclonal anti-type 3 deiodinase (D3; 1:2,000; Novus Biologicals, Littleton, CO), chicken polyclonal anti-glial fibrillary acidic protein (GFAP; 1:20,000; Abcam), and monoclonal anti-β-actin (1:10,000; Sigma, St. Louis, MO). After being rinsed in PBS-T, the blots were incubated for 1 hr at RT with either of the following horseradish peroxidase (HRP)-conjugated secondary antibodies in 2% BSA with 0.02% sodium azide: anti-rabbit IgG (1:5,000), anti-chicken IgY (1:5,000), and anti-mouse IgG (1:10,000; all from Jackson Immunoresearch, West Grove, PA). The blots were rinsed several times in PBS-T and visualized by exposure to Hyperfilm ECL (GE Healthcare, Buckinghamshire, United Kingdom) using an Immobilon Western Chemiluminescent HRP Substrate (Millipore). For quantification, the films were scanned, and the density of each band was measured in Image J (National Institutes of Health).

### Measurement of Iodothyronine Deiodinase Activity

Iodothyronine deiodinase activity was measured as previously described (Murakami et al., 1988), with minor modifications. To determine optimal conditions for the measurement of hippocampal D2 activity, iodothyronine deiodinase activity in the hippocampus of 1-month-old ICR mice was first characterized. Hippocampal samples were homogenized in homogenizing buffer (100 mM potassium phosphate, pH 7.0, containing 1 mM EDTA and 20 mM dithiothreitol) and centrifuged at 1,500*g* for 15 min at 4°C. The supernatants were incubated in a total volume of 50 μl containing various concentrations of [^125^I]T4 (NEN Life Science Products Corp., Boston, MA), which was purified using LH-20 (Pharmacia Biotech, Uppsala, Sweden) column chromatography on the day of experiment, 1 mM EDTA, 20 mM dithiothreitol, in the presence or absence of 1 mM 6-propyl-2-thiouracil (PTU) or 1 mM iopanoic acid for 2 hr at 37°C. The reaction was terminated by adding 100 μl ice-cold 2% BSA and 800 μl ice-cold 10% trichloroacetic acid. After centrifugation at 1,500*g* for 10 min at 4°C, the supernatant was applied to a small column packed with AG 50W-X2 resin (bed volume 1 ml; Bio-Rad Laboratories, Hercules, CA) and then eluted with 2 ml of 10% glacial acetic acid. Separated ^125^I was counted with a γ-counter. Nonenzymatic deiodination was corrected by subtracting I^−^ released in control tubes without homogenized samples. The protein concentration was determined by Bradford's method with BSA as a standard (Bradford, 1976). The deiodinating activity was calculated as fmol I^−^ released/mg protein/min, after multiplication by a factor of 2 to correct for random labeling at the equivalent 3′ and 5′ positions.

Each hippocampal sample from SAMP8 or SAMR1 was homogenized in 10 volumes of homogenizing buffer and centrifuged at 1,500*g* for 15 min at 4°C. Resultant supernatants were used for the measurement of D2 activity in the presence of 2 nM [^125^I]T4, 1 mM EDTA, 20 mM dithiothreitol, and 1 mM PTU, as described above.

### Immunohistochemistry

For immunohistochemical analysis, brain samples were fixed in 4% PFA for 48 hr at 4°C, dehydrated, embedded in paraffin, and cut into 8-μm-thick coronal sections. After deparaffinization and rehydration, the sections were incubated in 0.5% H_2_O_2_ for 20 min at RT to quench endogenous peroxidase activity, followed by 0.2% Triton X-100 for 15 min at RT. They were rinsed and incubated in 4% BSA for 30 min at RT to block nonspecific binding sites. Monoclonal anti-MBP antibody (1:1,000; Millipore) or monoclonal anti-S100 calcium binding protein B (S100β; 1:1,000; Sigma) was then applied to each section, and the sections were incubated overnight at 4°C. They were further rinsed and incubated with biotinylated secondary antibody (anti-mouse IgG; Chemicon/Millipore) at a dilution of 1:5,000 in BSA/PBS for 1 hr at RT. The sections were incubated in biotin-conjugated horseradish peroxidase with streptoavidin (ABC Elite Kit; Vector, Burlingame, CA) at a dilution of 1:1,000 for 30 min at RT and then visualized with nickel-intensified diaminobenzidine. The sections were counterstained with Carazzi's hematoxylin (Wako, Osaka, Japan), dehydrated and mounted in Entellan New (Merck, Darmstadt, Germany), and observed with a microscope (Axioplan 2; Carl Zeiss, Oberkochen, Germany).

### Statistical Analysis

All numerical data were expressed as mean ± SD and analyzed by one-way ANOVA. When ANOVA revealed significant differences, it was followed by two-tailed Student's *t*-test. The level of significance was set at *P* < 0.05.

## RESULTS

### Confirmation of the Age-Associated Cognitive Impairment in SAMP8 Mice

**Fig 1:**
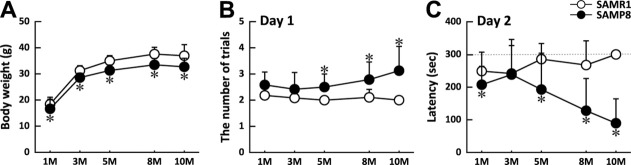
Body weights and cognitive performances of SAMP8 and SAMR1 mice between 1 and 10 months. **A:** Body weights of SAMP8 (solid circles) and SAMR1 (open circles). **B,C:** Comparison of learning and memory abilities of SAMP8 (solid circles) and SAMR1 (open circles) by passive avoidance test at different ages. On day 1 (B), number of trials required before each mouse learned to stay in the light compartment for 60 sec was counted. On day 2 (C), latency time before each mouse entered the dark compartment (up to 300 sec) was measured. Results are expressed as means + SD. A: n = 21–59 (SAMR1), n = 14–55 (SAMP8). B,C: n = 8–15 (SAMR1), n = 8–12 (SAMP8). *P < 0.05 between age-matched SAMP8 and SAMR1.

SAMP8 mice were active and showed little physical impairment during the observation period of up to 10 months. They had slightly lower body weights (8.5–11.5%) compared with the age-matched SAMR1 (Fig. .1A). Progressive decline in learning and memory abilities of SAMP8 mice was confirmed by passive avoidance conditioning as shown in Figure 1B,C. Average number of trials necessary for learning on the first day of trial was significantly greater in SAMP8 mice compared with SAMR1 at 5, 8, and 10 months of age (125.0% at 5 months, 126.6% at 8 months, and 156.3% at 10 months of age-matched SAMR1), whereas memory retention represented by the latency before entering the dark compartment on the second day was progressively decreased in SAMP8 mice between 5 and 10 months (67.5% at 5 months, 48.6% at 8 months, and 29.9% at 10 months of age-matched SAMR1). Both of these parameters remained constant in SAMR1 between 1 and 10 months, indicating that SAMP8 mice became progressively impaired in learning and memory starting at about 5 months.

### Behavioral Abnormalities of SAMP8 Mice Observed Prior to the Onset of Cognitive Decline

**Fig 2:**
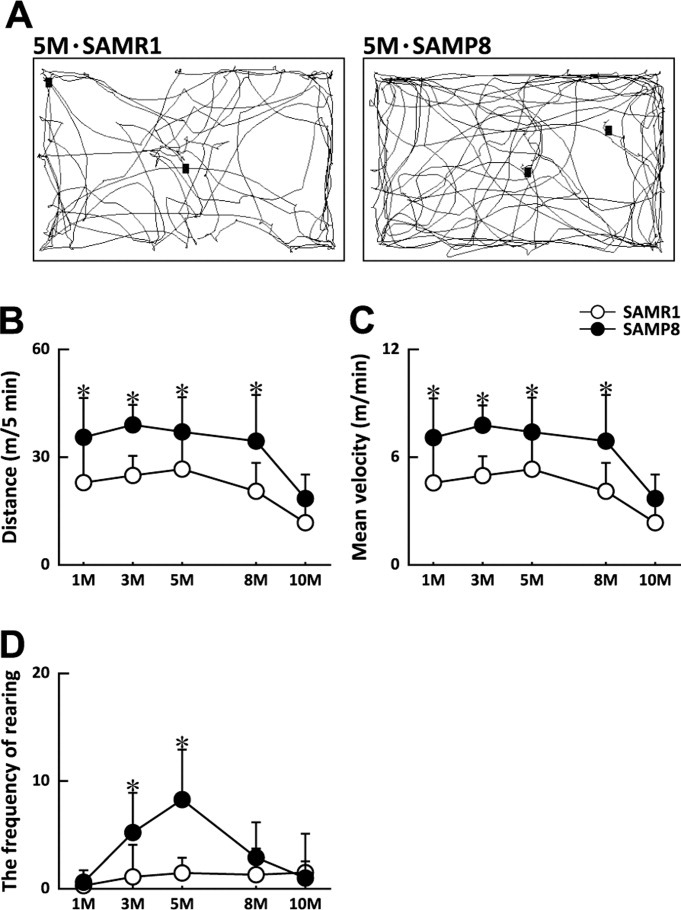
Behavioral comparison of SAMP8 and SAMR1 mice by open-field test at different ages. **A:** Typical examples of trajectories of 5-month-old SAMR1 and SAMP8 in the first 5 min in an unfamiliar environment. Total distance (**B**), mean velocity during movement (**C**), and the frequency of rearing in the central part of the open field (**D**), recorded during the 5 min of test session. Results are expressed as means + SD [n = 13–30 for 1–8 months, n = 8 for 10 months (SAMR1), n = 10–28 for 1–8 months, n = 5 for 10 months (SAMP8)]. *P < 0.05 between age-matched SAMP8 and SAMR1.

In addition to cognitive impairment, SAMP8 mice have been reported to exhibit behavioral abnormalities such as reduced anxiety that were also age dependent (Miyamoto et al., 1992; Markowska et al., 1998). With the open-field test, however, we noted hyperactivity and reduced anxiety in young SAMP8 mice before they started showing cognitive decline, as shown in Figure 2. Typical examples of trajectories during the first 5 min of the test session in an unfamiliar environment are shown in Figure 2A for SAMR1 and SAMP8 at 5 months. Measurement of total distance traveled during the first 5 min demonstrated a significant increase in SAMP8 mice at 1, 3, 5, and 8 months compared with SAMR1 (Fig. .2B). Mean velocity during movement was also higher by ∼30% in SAMP8 (Fig. .2C). In addition to such hyperactivity, rearing in the central part of the open field, interpreted as a sign of reduced anxiety, was frequently observed with SAMP8 mice at 3 and 5 months (Fig. .2D), whereas the frequency of rearing was minimal with SAMR1 at all ages.

### Differences in the Expression of Genes Involved in Local TH Metabolism and Signaling in the Hippocampus of SAMP8 and SAMR1 Mice

To examine the possible involvement of TH signaling in the cognitive impairment and abnormal behaviors of SAMP8 mice, we first compared plasma TH levels of SAMP8 and SAMR1. As shown in Table II, plasma levels of T4 and T3 determined by ELISA were similar in both strains at all ages examined (1–10 months), indicating that the systemic TH status was not altered in SAMP8.

**Table II:** Plasma Levels of Thyroxine (T4) and 3,5,3′-Triiodothyronine (T3) in SAMP8 and SAMR1 at Different Ages*

Age (months)	T4 (ng/ml)		T3 (ng/ml)	
SAMR1	SAMP8	SAMR1	SAMP8
1	103.1 ± 14.4	109.4 ± 12.2	9.9 ± 0.7	9.4 ± 1.2
3	115.0 ± 15.0	103.5 ± 10.4	10.4 ± 0.7	10.9 ± 0.1
5	103.6 ± 20.3	115.8 ± 17.1	10.7 ± 0.2	10.4 ± 0.4
8	118.8 ± 19.1	98.4 ± 11.2	10.3 ± 1.7	10.8 ± 0.1
10	83.8 ± 15.9	110.3 ± 9.2	10.9 ± 0.2	10.6 ± 0.4

* Values are expressed as means ± SD [n = 6 (SAMR1), n = 6 (SAMP8)]. Plasma levels of T4 and T3 were comparable in the two strains at all ages examined.

**Fig 3:**
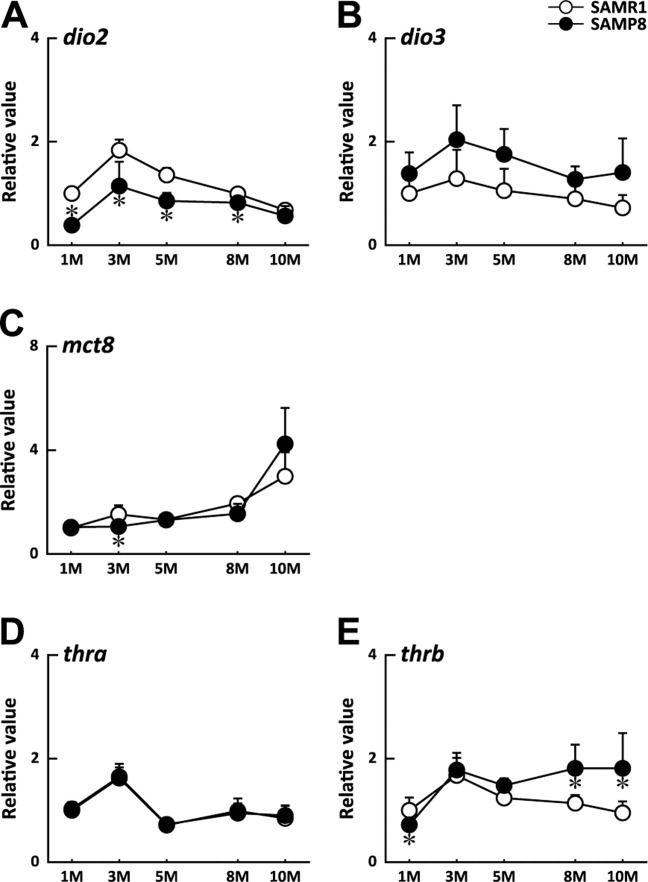
Comparison of expression profiles of genes involved in TH metabolism and signaling in the hippocampus of SAMP8 and SAMR1. The mRNA expression of TH-metabolizing enzymes (**A:***dio2*; **B:***dio3*), a TH transporter (**C:***mct8*), and TH receptors (**D:***thra*; **E:***thrb*) in the hippocampus of SAMP8 (solid circles) and SAMR1 (open circles) was quantified by real-time PCR at 1, 3, 5, 8, and 10 months using *ppia* as internal standard. Each mRNA level is expressed relative to that of SAMR1 at 1 month (means + SD of six mice/strain/age). *P < 0.05 between age-matched SAMP8 and SAMR1.

Because the level of transcriptionally active T3 in the CNS has been shown to be regulated locally, we next examined the expression of genes regulating local T3 availability as well as genes for its receptors in the hippocampus of SAMP8 and SAMR1 at different ages by real-time PCR. As shown in Figure 3A,B, significant differences between the two strains were observed in the mRNA expression of the two enzymes regulating the local T3 level. In the SAMP8 hippocampus, expression of the D2 gene (*dio2*), which converts T4 into active T3, was significantly downregulated at 1, 3, 5, and 8 months (Fig. .3A), whereas that of the D3 gene (*dio3*), responsible for inactivation of T3, showed a tendency to be upregulated (Fig. .3B) in comparison with SAMR1. The major neuronal TH transporter, monocarboxylate anion transporter 8 (*mct8*), was also significantly downregulated in SAMP8 at 3 months (Fig. .3C).

The mRNA expression of TH receptor α (*thra*) was similar in both strains at all time points (Fig. .3D), whereas TH receptor β (*thrb*) was significantly downregulated at 1 month and upregulated at 8 and 10 months in SAMP8 (Fig. .3E). Both of these TH receptors as well as *dio2* and *dio3* showed a peak of expression at 3 months, suggesting the importance of TH signaling at the maturation stage.

### Reduction in D2 Protein and D2 Activity in the Hippocampus of SAMP8

**Fig 4:**
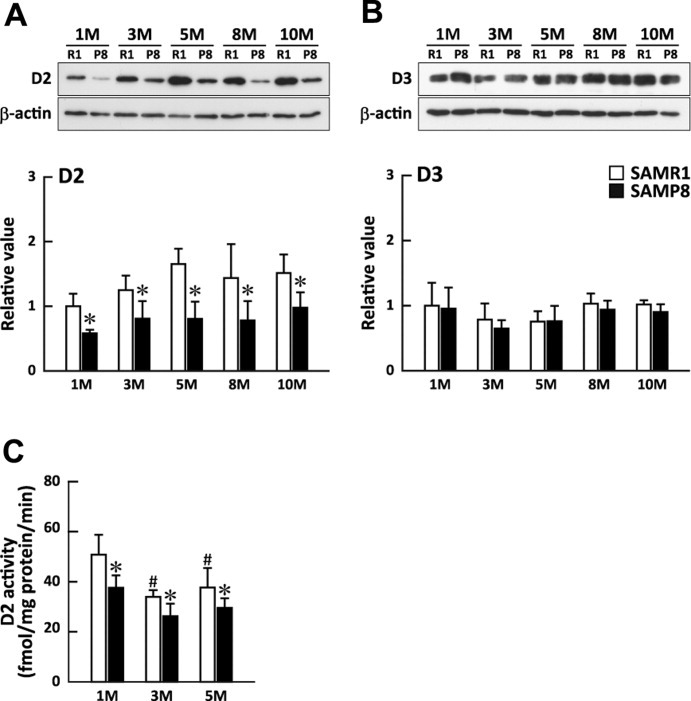
D2 and D3 protein levels and D2 activity in the hippocampus of SAMP8 and SAMR1 at different ages. D2 (**A**) and D3 (**B**) protein levels quantified by Western blotting with anti-D2 and anti-D3 antibodies, respectively. Immunoreactive bands of D2 and D3 (examples shown in upper panels) in the hippocampus of SAMP8 (solid bars) and SAMR1 (open bars) at different ages were quantified using β-actin as standard and expressed relative to that of SAMR1 at 1 month. Results are expressed as means + SD [n = 6–8 (SAMR1), n = 5–6 (SAMP8)]. *P < 0.05 between age-matched SAMP8 vs. SAMR1. **C:** D2 enzyme activity in the hippocampus of SAMP8 (solid bars) and SAMR1 (open bars) at 1, 3, and 5 months expressed as means ± SD [n = 6 (SAMR1), n = 5–6 (SAMP8)]. *P < 0.05 between age-matched SAMP8 and SAMR1. ^#^*P* < 0.05 compared with the value at 1 month of the same strain.

Sustained reduction of *dio2* mRNA between 1 month and 5 months resulted in 35–50% reduction of the D2 protein in the hippocampus of SAMP8 compared with SAMR1 throughout the observation period, as shown in Figure 4A. In contrast, there was no difference in D3 protein level between the two strains at all time points examined (Fig. .4B).

To compare further the D2 enzyme activity in the hippocampus of SAMR1 and SAMP8, iodothyronine deiodinase activity was first characterized with tissues from adult ICR mice. Hippocampal T4 deiodinating activity was not influenced by 1 mM PTU but was completely inhibited by 1 mM iopanoic acid. From the double reciprocal plot, kinetic constants for T4 were calculated to be K_m_ = 4.7 nM and V_max_ = 68 fmol I^−^ released/mg protein/min. These characteristics of iodothyronine deiodinase activity were compatible with D2 activity.

In the SAMR1 hippocampus, D2 activity was highest at 1 month and significantly decreased (21–29%) with maturation of the animal at 3 and 5 months. Compared with that in SAMR1, hippocampal D2 activity was confirmed to be reduced by 21–23% in SAMP8 at 1, 3, and 5 months (Fig. .4C).

### Differences in the Expression of TH-Responsive Genes in the Hippocampus of SAMP8 and SAMR1 Mice

**Fig 5:**
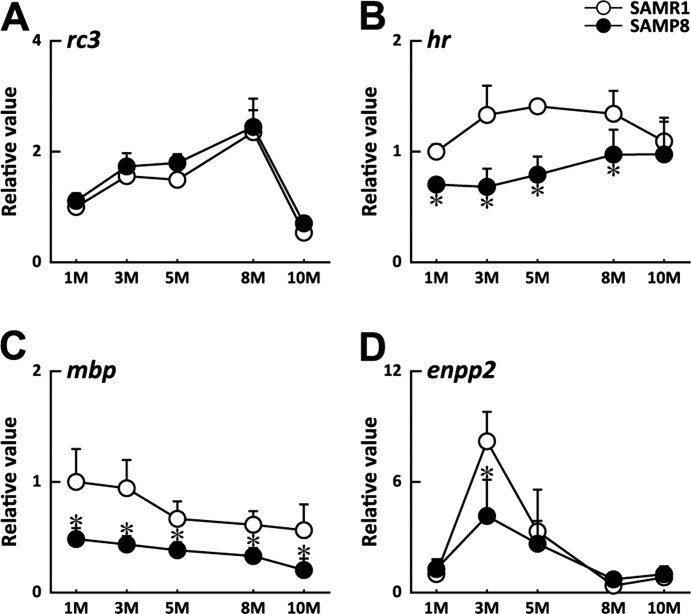
Comparison of expression profiles of TH-responsive genes in the hippocampus of SAMP8 and SAMR1. The mRNA expression of *rc3* (**A**), *hr* (**B**), *mbp* (**C**), and *enpp2* (**D**) in the hippocampus of SAMP8 and SAMR1 was quantified by real-time PCR at 1, 3, 5, 8, and 10 months using *ppia* as internal standard. Each mRNA level is expressed relative to that of SAMR1 at 1 month (means + SD of six mice/strain/age). *P < 0.05 between age-matched SAMP8 and SAMR1.

To determine whether TH signaling was affected in the SAMP8 hippocampus as suggested by the reduction in the availability of active T3, we next examined the mRNA expression of the following four known TH-dependent genes; neurogranin (*rc3*), myelin basic protein (*mbp*), hairless (*hr*), and ectonucleotide pyrophosphatase/phosphodiesterase 2 (*enpp2*). These genes are either equipped with the TH-responsive element (TRE) or have a great possibility of possession of TRE (Farsetti et al., 1991; Martínez de Arrieta et al., 1999; Potter et al., 2002; Freitas et al., 2010). Although the mRNA expression of *rc3* was similar in both strains (Fig. .5A), expression of *hr* at 1, 3, 5, and 8 months and *mbp* at all time points and of *enpp2* at 3 months were significantly downregulated in the hippocampus of SAMP8 compared with SAMR1 (Fig. .5B–D).

### Immunohistochemical Analysis of MBP Expression in the Hippocampus of SAMP8 and SAMR1 Mice

**Fig 6:**
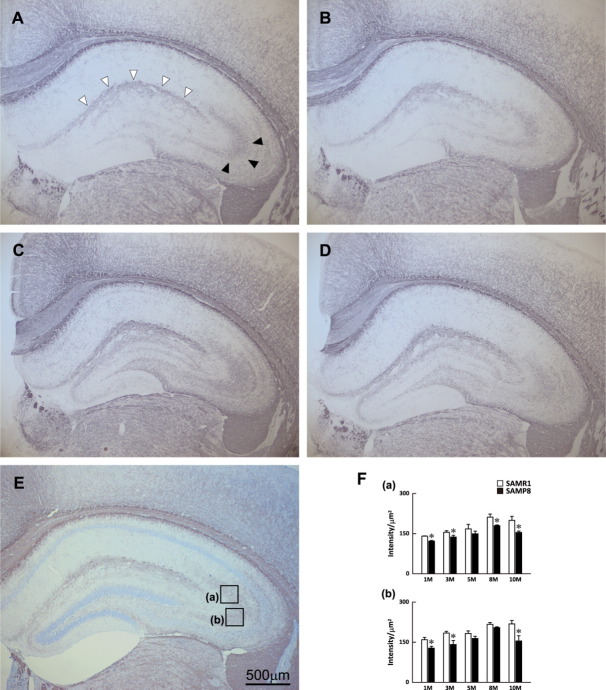
Immunostaining with anti-MBP antibody in the hippocampus of SAMP8 and SAMR1 at different ages. Hippocampal sections (8 μm thick) from SAMP8 and SAMR1 aged 1, 3, 5, 8, and 10 months were stained with anti-MBP antibody. **A–D** show sections from SAMR1 (A) and SAMP8 (B) at 1 month and SAMR1 (C) and SAMP8 (D) at 10 months. Open arrowheads, stratum lacunosum-moleculare; solid arrowheads, stratum radiatum of CA3. In **E**, a section from SAMR1 at 5 months counterstained with hematoxylin is shown. **F:** Total intensity of MBP immunoreactivity in two defined areas in CA3 (represented by the two squares in E) was quantified individually at different time points. Results are expressed as means + SD. Three sections per mouse and three mice/strain/age were analyzed. *P < 0.05 between age-matched SAMP8 and SAMR1.

Because the mRNA expression of *mbp* in the SAMP8 hippocampus was significantly downregulated at all time points, we examined the expression of MBP protein by immunohistochemistry. As shown in Figure 6, prominent MBP immunoreactivity was observed in two regions of the hippocampus of both strains at all time points examined, one running along the stratum lacunosum-moleculare (open arrowheads in Fig. .6A) and the other running through the stratum radiatum of CA3 in a crescent shape (solid arrowheads in Fig. .6A). The former corresponds to the myelinated entorhinal projections (perforant path), and the latter corresponds mainly to the Schaffer collaterals, both of which are most intensively stained by other staining methods specific for myelin (Meier et al., 2004). The staining intensity appeared lower in SAMP8 compared with SAMR1 in these regions, especially at 1 month (Fig. .6A,B) and at 10 months (Fig. .6C,D). Quantification of total intensity of MBP immunoreactivity in two defined areas in the CA3 stratum radiatum (represented by squares a and b in Fig. .6E) at different time points showed a gradual increase in intensity up to 8 months in both strains (Fig. .6 F). Compared with that in SAMR1, MBP staining in SAMP8 was lower at 1, 3, and 8 months (Fig. .6F), suggesting a slower progression of myelination in the young SAMP8 mice, which never reached the level of SAMR1 at its maximum (8 months). Furthermore, a significant reduction in MBP intensity observed only in SAMP8 at 10 months indicates an earlier onset of age-related myelin loss in SAMP8.

### Distribution and Morphology of Astrocytes in the Hippocampus of SAMP8 and SAMR1

**Fig 7:**
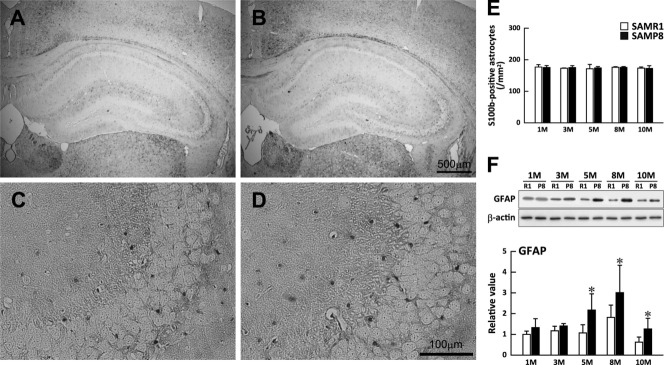
Astrocyte density and GFAP content of the SAMP8 and SAMR1 hippocampus at different ages. Astrocyte cell bodies were visualized by immunostaining with anti-S100B antibody in the whole hippocampus (**A,B**) and CA3 region (**C,D**) of SAMR1 (A,C) and SAMP8 (B,D) mice at 3 months. **E:** Number densities of astrocytes in the whole hippocampus of SAMR1 (open bars) and SAMP8 (solid bars) were obtained by counting S100B-positive cell bodies at different ages and expressed as mean + SD [n = 3–8 (SAMR1, SAMP8)]. **F:** GFAP content of the SAMR1 (open bars) and SAMP8 (solid bars) hippocampus quantified by Western blotting with anti-GFAP antibody. Immunoreactive bands of GFAP (examples shown in the upper panel) at different ages were quantified using β-actin as standard and expressed relative to that of SAMR1 at 1 month. Results are expressed as means + SD [n = 5–8 (SAMR1), n = 5–6 (SAMP8)]. *P < 0.05 between age-matched SAMP8 and SAMR1.

Because D2 is localized to astrocytes, we further examined whether a reduction in D2 protein and activity simply reflected a decrease in astrocyte population or not. By immunohistochemical staining of astrocytic cell bodies with S100β (Fig. .7A–D), the number density of astrocytes in the hippocampus of the two strains was found to be similar and constant between 1 month and 10 months (Fig. .7E). In contrast, the amount of GFAP was significantly increased in SAMP8 at 5, 8, and 10 months compared with SAMR1 (Fig. .7F), in accordance with the morphological changes into reactive astrocytes (data not shown) as reported previously for aged SAMP8 mice (Nomura et al., 1996).

## DISCUSSION

### Altered Local T3 Signaling in the Hippocampus of the Young SAMP8 Mouse

In the present study, we show for the first time significant alterations in the expression profiles of TH-metabolizing enzymes in the hippocampus of young SAMP8 compared with SAMR1. In SAMP8, large reductions in the *dio2* mRNA level were observed between 1 month and 5 months, resulting in 35–50% reduction in the D2 protein level throughout the observation period (Figs. .3, 4) and significant reductions in D2 activity at 1, 3, and 5 months (21–23%). On the other hand, D3 protein level was similar in both strains and was constant between 1 and 10 months.

Recent studies have established that TH signaling is precisely regulated locally by D2 and D3 activities (Dentice and Salvatore, 2011). The T3-degrading enzyme D3 is expressed mainly in fetal tissues, in which unoccupied TH receptors generally maintain cell proliferation and prevent premature differentiation (Williams, 2008). A rapid rise in T3 in mammals at birth is brought about by concomitant downregulation of *dio3* and upregulation of *dio2*. Postnatal expression of *dio3* is restricted to only a few tissues, including the brain, in which it is localized to neurons (Williams, 2008). Significant reduction in D2 protein and D2 activity with constant level of D3 protein observed in the present study thus suggests a decrease in the local availability of transcriptionally active T3 in the young SAMP8 hippocampus. Although it was not possible to assess the intracellular concentration of T3, weaker TH signaling in the SAMP8 hippocampus was confirmed by more than two-fold reductions in the expression of the three genes directly regulated by T3, especially at 3 and 5 months, when behavioral abnormalities of SAMP8 were most pronounced. Expression of one TH-responsive gene, *RC3/neurogranin*, was not altered in SAMP8, probably because this gene for the postsynaptic protein is abundantly expressed only between 1 and 2 weeks postnatally.

Further studies are needed to determine the causes of the reduction of D2 protein and D2 activity in SAMP8 mice. D2 is an endoplasmic reticulum (ER)-resident protein in astrocytes and tanycytes regulated posttranslationally by ubiquitination-dependent inactivation and degradation, which are enhanced by T4 (for review see Arrojo e Drigo and Bianco, 2011). Because the densities of S100B-positive astrocytes were similar and constant with age in both strains and GFAP immunoreactivity in the SAMP8 hippocampus was comparable to that in SAMR1 up to 3 months (Fig. .7), we can rule out the possibility that the reduction in D2 protein simply reflects a decrease in astrocyte population. At the transcriptional level, *dio2* has been shown to be downregulated by oxidative stress and inflammation, whereas *dio3* is upregulated (Lamirand et al., 2008; Simonides et al., 2008; Calzà et al., 2010). Such reciprocal changes in the expression of *dio2* and *dio3* in response to pathological conditions lead to reduced local T3 concentration, which in turn should decrease metabolic activity and prevent further production of oxidative stress. Reduced expression of *dio2* in SAMP8 at older ages may arise from elevated oxidative stress (Butterfield et al., 1997; Tomobe et al., 2012) or chronic expression of proinflammatory cytokines such as interleukin (IL)-1β, IL-6, and tumor necrosis factor-α (Tha et al., 2000) reported for SAMP8 at 10 months. On the other hand, an explanation for the severe downregulation of *dio2* at earlier time points (1 and 3 months) awaits further studies on the perinatal development of the TH system of SAMP8.

### Delayed Myelination Followed by Earlier Onset of Myelin Degeneration in the SAMP8 Hippocampus

In addition to signs of oxidative stress (Butterfield et al., 1997) and increased amyloid β burden (Del Valle et al., 2010), loss of myelin and myelinating oligodendrocytes have been previously reported for the brains of old SAMP8 (10 months of age; Tanaka et al., 2005). The present study reveals that there is already an impairment of myelination during development.

It is well established that TH plays a critical role in developmental myelination, both through regulation of oligodendrocyte differentiation and through expression of myelin component genes such as *mbp* (Oppenheimer and Schwartz, 1997; Koibuchi and Chin, 2000). Importance of TH in remyelination in the adult CNS has also been highlighted in recent studies on inflammatory-demyelinating diseases in which immune activation and brain injury downregulate *dio2* and lead to a decrease in local T3 availability (Calzà et al., 2005, 2010). A decrease in MBP immunoreactivity observed in SAMP8 at later time points (10 months) may result from downregulation of *dio2* by oxidative stress and/or by proinflammatory cytokines.

### Possible Relationship Between TH Deficiency and Abnormal Behavior of Young SAMP8 Mice

In addition to the age-associated cognitive impairment, behavioral abnormalities of SAMP8 mice have been described in an earlier study by Miyamoto et al. (1992), in which the authors detected lower anxiety in an elevated plus maze test, food neophobia, and punished drinking test. These behavioral characteristics were noted at 4 months in SAMP8 and became progressively more pronounced with age. Because a reduction in anxiety was also found in SAMR1 at a much older age (20 months), it was concluded that behavioral abnormalities of SAMP8 was an earlier manifestation of age-related emotional disorders. By comparing SAMP8 and SAMR1 at 4 months and 15 months, Markowska et al. (1998) also noted reduced anxiety by plus maze test, hyperactivity by open-field test, and an age-related impairment of sensorimotor performance.

Our study on the behavior of the two strains at five time points between 1 month and 10 months clearly demonstrated that hyperactivity and reduced anxiety were not aging-dependent behavioral abnormalities but were characteristic of young SAMP8 before the onset of cognitive decline. Reduced TH signaling resulting from downregulation of *dio2* overlaps in timing with behavioral alterations, suggesting a possible link between the two phenomena.

Two lines of evidence relate the developmental deficiency in TH signaling to attention deficit-hyperactivity disorder (ADHD) in humans. One is the high incidence of ADHD (40–70%) associated with generalized resistance to thyroid hormone (RTH), a rare genetic syndrome caused by mutations in the TH receptor β (TRβ) gene that result in reduced T3 binding (Hauser et al., 1993). RTH is characterized by elevated serum T4 and T3 concentrations, accompanied by normal or elevated level of thyroid-stimulating hormone (TSH) and by reduced responsiveness of the pituitary and peripheral tissues to TH. Knock-in mice expressing a human mutant TRβ allele (TRβPV) found in RTH exhibited hyperactivity, impaired learning, and altered responsiveness to methylphenidate resembling human ADHD (Siesser et al., 2005, 2006).

The second piece of evidence linking TH and ADHD comes from studies on preterm infants (Simic et al., 2009). Because the fetal thyroid does not produce significant amounts of TH until the third trimester in humans (Williams, 2008), most preterm infants experience a transient neonatal hypothyroidism during what should have been the later part of pregnancy in which maternal TH supply is still important. TH insufficiency is thus considered a major cause of neurocognitive deficits with reduced attention at 3 months of age (Simic et al., 2009). Attention-deficit and hyperactive behavior could also be reproduced in an animal model of perinatal hypothyroidism obtained by administering PTU to pregnant rats during pregnancy and lactation periods (Negishi et al., 2005). Although body weights at birth were comparable to those of euthyroid pups, offspring of PTU-treated dams showed a reduction in postnatal body weight increase. When tested behaviorally at the young adult stage (8 weeks of age), they exhibited hyperactivity, low anxiety, impaired learning, and shortened attention span compared with euthyroid animals.

Although SAMP8 mice have been considered as models of age-dependent cognitive disorders, our study shows that, at younger ages, they may serve as an interesting model of developmental disorders caused by local T3 deficiency. The present results also raise the possibility that accelerated senescence in SAMP8 mice may originate from subclinical deficits in TH signaling during development. Perturbations in the TH system can occur not only endogenously but also through environmental factors such as iodine or Se deficiency and intake of some chemicals (Takahashi et al., 2009), so studies on SAMP8 both at younger and at older ages should provide valuable insights into the role of the TH system in the development and maintenance of the CNS.
